# Microgrids: A Model for Basic Microsurgery Skills Training

**DOI:** 10.5704/MOJ.1807.007

**Published:** 2018-07

**Authors:** J Gunasagaran, RJ Rasid, S Mappiare, C Devarajooh, TS Ahmad

**Affiliations:** Department of Orthopaedic Surgery (National Orthopaedic Center of Excellence for Research & Learning - NOCERAL), University of Malaya, Kuala Lumpur, Malaysia; ^*^Department of Orthopaedics, Newcastle University Medicine Malaysia, Iskandar Puteri, Malaysia; ^**^Department of Social Preventive and Medicine, University of Malaya, Kuala Lumpur, Malaysia

**Keywords:** microgrid, microsurgery training

## Abstract

**Introduction:** Microsurgery is a subspecialised field which requires high technical skill. Laboratory training offers good opportunity for novice surgeons to learn and repetitively practise their skills prior to hands-on clinical practice. Commonly, the training programme consists of models in a stepwise increase in fidelity: from latex sheet to anaesthetised rat. We introduce microgrids model as a daily warm up procedure in a 5-day basic microsurgery course. The purpose of this study is to evaluate the correlation between microgrids colouring under magnification with microsuturing proficiency among novice surgeons.

**Materials and Methods:** Participants were required to fill in microgrids under magnification everyday during their 5-day training as a starter test. The number of completely filled in microgrids in 20 seconds was recorded. A simulated cut on latex sheet was sutured and the time taken to apply five sutures was recorded. The sutures were evaluated with modified Global Rating Scale (GRS). Data was analysed with SPSS.

**Results:** There was a statistically significant correlation between the number of microgrids coloured and the time taken to apply five sutures (p<0.01). An increase in number of microgrids coloured was significantly associated with the increase in quality of the suturing technique (p< 0.01). During the 5-day basic microsurgery skills training for novice surgeons, microsuturing skill improvement correlated with microgrid colouring.

**Conclusion:** Microgrids colouring reflected microsuturing proficiency. It is an inexpensive, readily available, and simple model of ‘warm up’ for hand dexterity. The microgrids model can function as a starter test for initial training and a quick screening measure to assess microsurgical skill.

## Introduction

Microsurgery is a fundamental technique for complex reconstructions today^1^. The success or failure of a free tissue transfer reconstruction is dependent upon maintaining arterial inflow and venous outflow through a patent anastomosis, and this has been recognised widely to be operator-related. A microsurgeon needs to equip him- or herself with microsurgical knowledge, decision-making, time management and communication skills^2^. The most important component in competency is technical surgical performance in microsurgery which cannot be acquired from mere observation. This needs repetitive training and most surgeons would be trained at a standard microsurgery course or workshop.

The medical profession is constantly under pressure to develop methods to produce competent surgeons^3^ and assure healthcare quality. An adequate training curriculum is necessary to ensure that microsurgical techniques are practised well, and to reduce or avoid extreme variation in surgical competence. Microsurgical techniques can be trained in the laboratory using simulation as this provides a good opportunity for young surgeons to practise initial basic microsurgical skills prior to hands-on clinical practise on patients. In a 5-day laboratory workshop training, 60% of trainees had improved in the level of microsurgery skill^4^.

Training curriculum must precede assessment in order to provide comprehensive feedback as part of the learning process, and to measure improvement over time. An ideal assessment tool must be valid, reliable, cost-effective, and feasible^2^ and that it should not add a significant amount of time to the workplace task. With expansion and evolution of microsurgery training, many models are introduced for training and assessment purposes. We developed a structured training scheme and studied the value which microgrids model has as a ‘warm up’ tool in dexterity training and screening measure in training progress for basic microsurgery skills.

The objective of this study is to evaluate the correlation between microgrids colouring under magnification with proficiency of microsuturing among novice surgeons during the 5-day basic microsurgery skill training.

## Materials and Methods

This was a cross-sectional study conducted in the Microsurgery Training & Research Laboratory, NOCERAL, University Malaya from June 2015-June 2016. Twenty four medical officers were recruited. Twenty participants were registrars in Orthopaedic department and four were medical officers from district hospitals. None of them had exposure in microsurgery prior to this course. A brief introduction to the microinstruments and microscope demonstration was given.

**Fig. 1:**
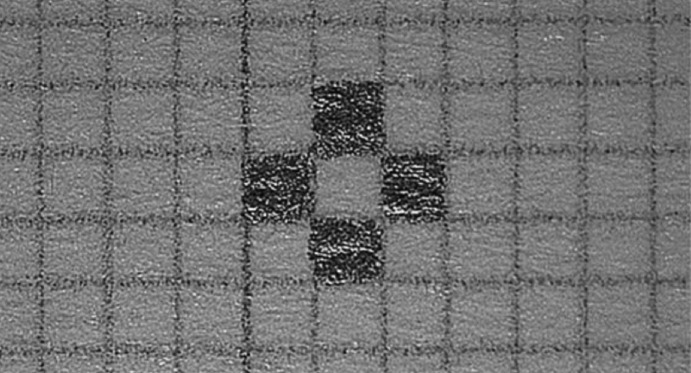
Microgrids model as a microsurgery starter test.

The methodology contained two main assessments: the microsurgery starter and the suture proficiency tests. For the microsurgery starter, participants were given 20 seconds to perform a simple exercise under a microscope which involved filling in as many small boxes measuring 1x1mm (microgrid) as possible on a graph paper with a mechanical pencil. Each microgrid was required to be filled in completely without any defects. The microgrids had to be coloured alternately and the surrounding boxes were to be left blank (Fig. 1).

**Fig. 2:**
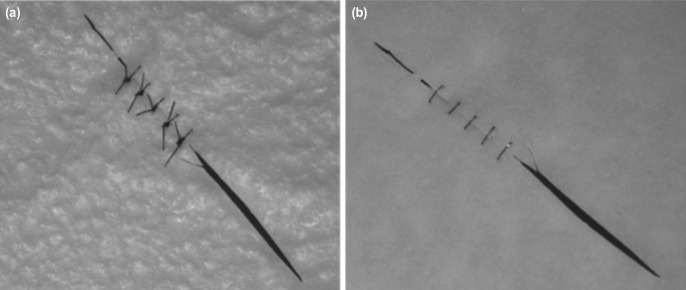
(a) Upper surface of latex sheet showed the 5 knots and (b) Undersurface of the same latex sheet showed parallelity of sutures, approximation of cut edges of latex sheet, proper suture spacing and placement.

Once completed, the participants were instructed to perform the suture proficiency test, whereby they were instructed to simulate a laceration on a latex sheet and repair the cut. These were done by placing five sutures (Nylon 9-0) at 1mm intervals. Time was recorded from the start of first suture to the end of the fifth suture. Both the tests were repeated and performed by the participants at the start of each day of the 5-day Basic Microsurgery Course. The number of microgrids coloured and the time taken to complete five sutures were recorded everyday under supervision of the course facilitator (Fig. 2).

The microgrids and latex sheets were then evaluated by a blinded microsurgeon to assess the quality of the microgrids coloured and sutures applied. Microgrids with either defective or incomplete colouring were excluded. Number of perfect microgrids were recorded. Sutures were checked for proper spacing (1mm), quality of knot, approximation of the cut edges, distance of suture end from cut edge and parallelity of sutures. A modified Global Rating Scale (GRS), 5-point scale was used to score each entity: 1 for very poor, 3 for moderate and 5 for very good. All scores were added, a minimum of 5 and maximum of 25, and recorded each day of the test.

Data was analysed with SPSS statistical analysis software (SPSS Inc. Chicago). The one-way analysis of variance (ANOVA) test was done for each component to assess difference in performance within the five days of training. Post-hoc comparisons were done with Bonferroni with significance level of p-value less than 0.05. Relationship between the number of microgrids coloured in 20 seconds, time taken to complete five sutures and the quality of suturing (GRS) were evaluated with Pearson’s correlation coefficients (2-tailed). P-value of less than 0.01 was used as statistical significance level.

## Results

The number of microgrids on the graph paper that was coloured within 20 seconds by the participants gradually increased during the 5-day course. The mean number of microgrids filled in on Day 1 was seven and gradually increased to 16 on Day 5 of training. The one-way ANOVA test showed a statistically significant difference between first day of colouring compared to other days (p<0.05). Post-hoc comparison with Bonferroni showed significant increase in number of microgrids between Day 1 of colouring and each of other subsequent days (p<0.05) except Day 2. The participants’ performance improved significantly with each progressing days of training. However, there was no significant difference between Day 1 and Day 2 of microsurgery training.

The time taken to complete five sutures with 1mm interval decreased gradually during the five days of microsurgery skills training. On the first day, average duration was 14 minutes which decreased gradually to nine minutes on Day 5 of training. This showed that the participants were able to suture faster with progressive days of training. There was a significant improvement between Day 1 and Day 5 (p< 0.05).

**Fig. 3:**
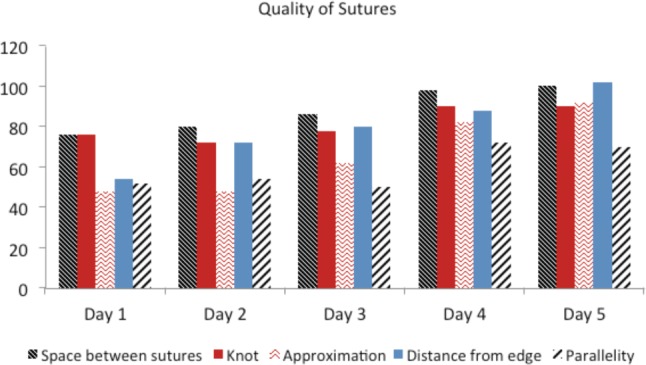
Global Rating Scale (GRS) to assess quality of suturing.

The quality of the suturing had improved significantly between Day 1 and Day 4 to Day 5 (p< 0.05). Mean total score on Day 1 was 12.8 whereas on Day 5 was 18.9. There was a gradual increase according to the Global Rating Scoring (Fig. 3).

**Fig. 4:**
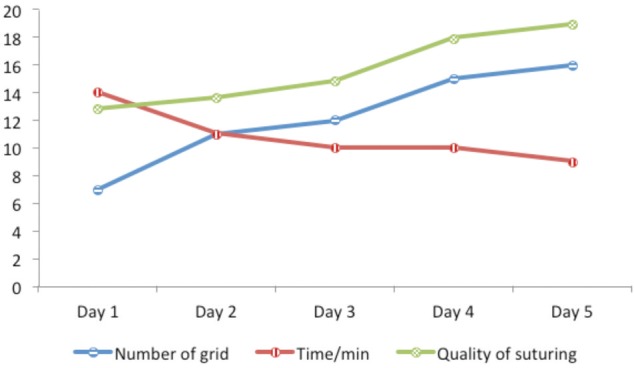
Correlation between microgrids, time (min) and quality of suturing technique (GRS).

The number of microgrids filled in was linked to the duration taken to complete the five sutures and the quality of the suturing. As the number of microgrids coloured increased with progressive days of training, the time taken to apply the five sutures decreased and the quality of suturing increased. There was statistically significant correlation, Pearson (2-tailed) correlation of -0.268 between colouring microgrids and time taken to apply five sutures (p<0.01). An increase in the number of microgrids coloured was significantly associated with the quality of the suturing technique, Pearson correlation 0.348 (p< 0.01) (Fig. 4).

## Discussion

Microsurgery is a fine and complex field, frequently practised in reconstruction in plastic surgery, hand surgery, and oral and maxillofacial surgery. It requires high technical skills inclusive of ability in decision-making, time management and many other attributes. The knowledge can be gained by reading and attending didactic courses. However, the technical skills can only be attained, maximised and retained from repetitive practical training^5^. Ethan *et al* found that the outcome of training in hands-on practise with model simulations were significantly better compared to didactic training^5^.

Surgical skills training were challenging in the past as the trainees were under constant pressure from their consultants or senior surgeons to produce competent apprentices. In the current situation, an increase in the number of surgeons prevents adequate chance to practise microsurgery. The training skills are cumbersome to be individually assessed. Futhermore, the consultants frequently face medicolegal issues pertaining to patient care. It is inappropriate and unethical to allow a trainee to start first hands-on practice on patients when capability is questionnable. A logical alternative would be laboratory-based training in order to improve basic skills and techniques. It is an opportunity for the trainee to prepare himself adequately prior to performing surgery in real operating theatre situations. Even trained microsurgeons have to undergo laboratory training to refine their skills^4^.

With advances in microsurgery training, many models are now available. The gold standard, undeniably, remains the femoral vessels of anaesthetised rats which are 3-dimensional models and can be used to assess tissue dissection, adventitial stripping and blood flow^6^. However, there are also ethical issues on use of animals in research or training and costs required in maintenance of the animal labarotary. Some studies recommend non-living models like rubber practise pad, surgical gauze, silicone tubes, glove box model and foliage leaf to function as a complement to living models^3^. Many make an effort to improve these models in order to achieve higher fidelity. Lahiri *et al* suggested suturing latex sheet models with an ‘I’ and ‘double triangle’ cut which simulates vessel wall suturing mechanically^7^. The ‘round-the-clock’ model is 3-dimensional and recommended as a warm up exercise as well as an instant assessment tool^8^. Virtual reality simulators are current favourite tools in training endoscopic and minimal invasive surgeries^9^, and may replace living models in microsurgery training in the future^6^. They are costly and not easily available. A stepwise method is introduced in microsurgery skill training with gradual increase in fidelity. One should practise on non-living models and progressively advance to living models before managing patients in clinical settings. However, the level of bench model fidelity does not affect the technical skills gained by trainees^10^. Whichever models are used in laboratory training, the microskills learned were transferred and applied efficiently in clinical practice in the operating theatre.

In our centre, we organise a basic microsurgery skills training course which is compulsory for our registrars prior to their microsurgery procedures on patients. The 5-day course includes the microsurgery starter test (microgrids model) for dexterity, suturing latex sheet, vessel anastomosis in chicken wings^11^ and thighs followed by anaesthetised rats.

We introduced a new method in improving and training hand dexterity in microsurgery. Perfectly colouring microgrids under magnification requires a lot of patience and stable hands. From our study, we proved that the number of microgrids filled improved with repetitive practise. In addition, we found that participants were able to complete sutures faster and the quality of suturing improved gradually with training. There was significant correlation between the number of microgrids filled with speed and quality of suturing. Thus, we recommend this model to be used as a ‘starter’ or warm up prior to the suturing sessions. The focus of the microgrids model is to train basic skills on hand dexterity, handling the microscope, familiarising with magnified vision and instill patience in performing micro-procedures. Indirectly, it can also predict the capability of a surgeon in microskills. Therefore, it is a simple, cost-effective method in quick assessment of microskills despite being a 2-dimensional model. In future, this method can also be further refined through constructive feedback.

There were limitations in this study. The temporal relationship between microgrids colouring and microsurgery skills could not be determined.

Assessment of training is important in evaluating the outcome of a microsurgery training course. It determines if the course has achieved its objective in producing competent microsurgeons and its constructive feedback important in improving the learning curve^12^. Logbooks and clinical observations are too subjective to quantify the skill achieved by the trainees. Structured assessment of microsurgery skills (SAMS) is an objective method of assessment which comprises modified Global Rating Score (GRS), errors list and summative rating^13^. In ophthalmic surgery, a similar method was used which is video-based modified Objective Structured Assessment of Technical Skill (OSATS)^14^. We used clinical observation with criteria, a reliable and valid objective assessment tool^2^ in evaluating the number of microgrids coloured and the quality of sutures applied on latex sheet. GRS was used to grade the quality of sutures according to the important factors that determines the success of vessel anastomosis.

## Conclusion

During the 5-day basic microsurgery skills training for novice surgeons, microsuturing skills improvement, inclusive of speed of suturing and quality of sutures applied, correlated with microgrids colouring under magnification. Thus, microgrids colouring reflected the microsuturing skills. It is an inexpensive, readily available, and simple model for ‘warm up’ in a stepwise training programme to practise rudimentary skills and hand dexterity. We suggest the inclusion of microgrids model as a starter test for initial training of microsurgery skills before progress to high fidelity models in laboratory settings. In addition, it can be used as a quick screening measure to assess microskills.

## Conflict of Interest

The authors declare no conflict of interest.
